# Photochemistry of 1,4-Dihydropyridine Derivatives: Diradical Formation, Delocalization and Trapping as a Route to Novel Tricyclic and Tetracyclic Nitrogen Heterocyclic Ring Systems

**DOI:** 10.3390/molecules21070866

**Published:** 2016-06-30

**Authors:** Nader A. Al-Jalal, Yehia A. Ibrahim, Nouria A. Al-Awadi, Maher R. Ibrahim, Osama M. Sayed

**Affiliations:** Department of Chemistry, Faculty of Science, Kuwait University, P.O. Box 5969, Safat 13060, Kuwait; yehiaibrahim2002@yahoo.com (Y.A.I.); n.alawadi@ku.edu.kw (N.A.A.-A.); maherriyad@hotmail.com (M.R.I.); dr.osama_qc@yahoo.com (O.M.S.)

**Keywords:** photochemistry, 1,4-dihydropyridines, dihydropyrrolo[3’,4’4,5]cyclopenta[1,2-*b*]-pyridine, 4,11-diazatetracyclo[5,3,1,0^2,6^,0^8,10^]undecane, tetrahydropyrrolo[3’,4’:5,6]pyrano[4,3-*b*]-pyridine

## Abstract

Irradiation of an acetonitrile solution of 4-aryl-3,5-dibenzoyl-1,4-dihydropyridine derivatives **1a**–**c** and maleimides **2a**–**c** using medium pressure Hg-arc lamp (λ > 290) nm afforded three different cycloadducts **4**, **5**, **6** in addition to the oxidation products **3**. These results indicate that compounds **1a**–**c** undergoes intermolecular cycloaddition reaction through three biradical intermediates and behave photochemically different than those reported previously for the analogous 3,5-diacetyl and 3,5-dicarboxylic acid derivatives. The present work also offers simple access to novel tricyclic and tetracyclic nitrogen heterocyclic ring systems of potential biological and synthetic applications. The structure of the photoproducts was established spectroscopically and by single crystal X-ray crystallography.

## 1. Introduction

1,4-Dihydropyridines have historically played a very important role in the synthesis and mechanistic organic chemistry. Derivatives of 1,4-dihydropyridine (DHP) are drugs belonging to a class of pharmaceutical agents known as calcium channel blockers. They are inhibitors of calcium ion penetration inside cells and weaken the contractility of the cardiac muscle. These compounds have been shown to be very effective vasodilators and are useful in the treatment of hypertension, ischemic heart disease, and other cardiovascular disorders [1,2,3,4,5,6]. Among the most important representative of this group of drugs are nifedipine (NPDHP) and felodipine (CPDHP).

**Scheme 1:**
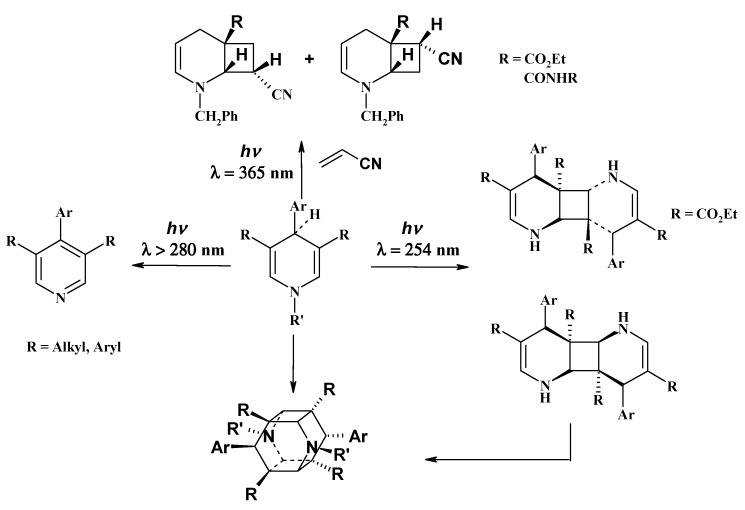
Reported photochemistry of 1,4-dihydropyridines.

Most photolytic reactions of 1,4-dihydropyridine (DHP) take place with elimination of molecular hydrogen followed by dimerization or aromatization products [7,8,9]. Moreover, Ponticelli reported that [10,11,12,13] photocycloaddition of acrylonitrile to 1-benzyl-1,4-dihydropyridine dicarboxylate derivatives afforded two diastereomeric azabicyclo[4.2.0]octane derivatives. Using *Z* or *E*-but-2-enenitrile in this photocycloaddition reaction gave products retaining the original alkene stereochemistry, thus proving the concerted nature of this [2 + 2] cycloaddition [13]. Also, 1,4-dihydropyridine dicarboxylate derivatives have been reported to undergo head-to-tail [2 + 2] photodimerization to give *anti*- and *syn*-dimers, and a cage dimer (formed by further intramolecular [2 + 2] photodimerization of the *syn*-dimer) (Scheme 1) [14,15,16,17].

In this work, we describe the photochemical behavior of 4-aryl-3,5-dibenzoyl-1,4-dihydropyridine derivatives **1a**–**c** and attempts to trap the possible intermediate diradicals with maleimides **2a**–**c**.

## 2. Results and Discussion

**Scheme 2:**
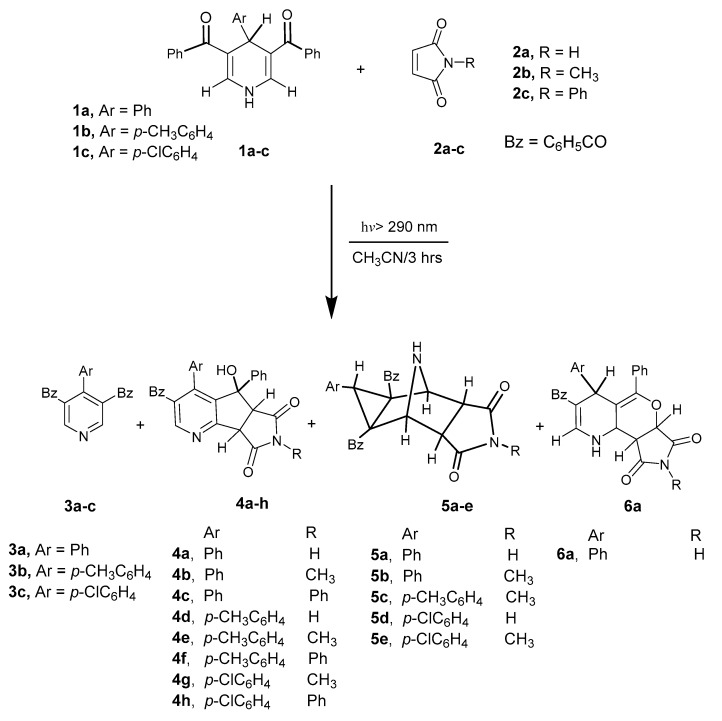
Photoproducts upon irradiation of 1,4-dihydropyridines **1a–c** with maleimides **2a–c.**

Several photoirradiation experiments were carried out with **1a** and **2a** in order to optimize the reaction conditions, involving changes of solvent (CH_2_Cl_2_, toluene, CHCl_3,_ and CH_3_CN), irradiation time, type of lamp (low or medium pressure Hg-arc lamps), the molar ratio of the two reactants (see Supplementary Materials). The optimum reaction conditions was achieved by using acetonitrile as a solvent, 3 h irradiation time, a 400 W medium pressure mercury arc-lamp (λ > 290 nm), a 1:3 molar ratio of 1,4-dihydropyridine **1a** with maleimide **2a** under a nitrogen atmosphere. These conditions were then applied to all photoreactions and the results are summarized in Table 1. Thus, irradiation of 1,4-dihydropyridines **1a**–**c** with maleimides **2a**–**c** using 400 W medium pressure mercury arc-lamp produced novel fused heterocyclic ring systems **4a**–**h**, **5a**–**e** and **6a**, together with the photooxidation products, the 3,5-dibenzoyl-4-aryl-pyridine derivatives **3a**–**c** (Scheme 2, Table 1).

**Figure 1:**
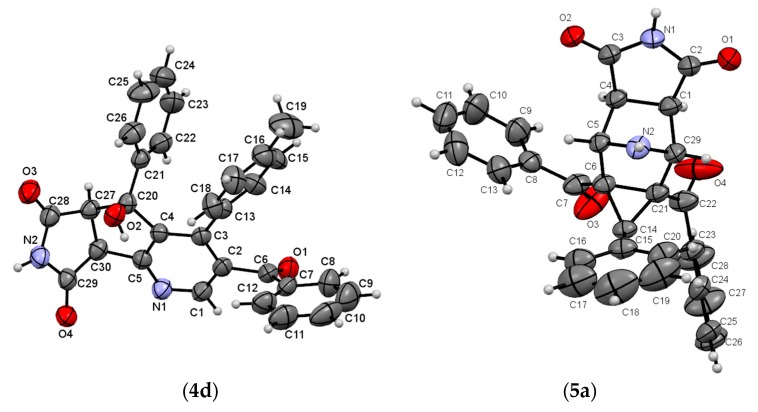

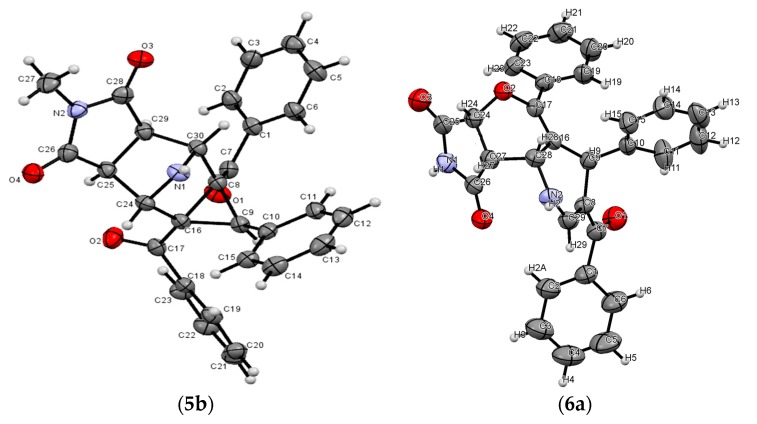
X-ray crystal structures of (**4d**), (**5a**)**,** (**5b**) and (**6a**).

The structure of all products has been established based on the ^1^H-NMR, ^13^C-NMR, HRMS and the X-ray crystal structures of photoproducts **4d**, **5a**, **5b** and **6a** (Figure 1). The ^1^H-NMR spectra of compounds **5a**–**e** showed interesting small coupling constants (*J* 0-2) for the vicinal ring protons (C-4, C-5 or C-1, C-29 shown in the X-ray of **5a**) consistent with a dihedral angle around 80° as found by the X-ray structure of two of these compounds **5a**,**b**.

**Scheme 3:**
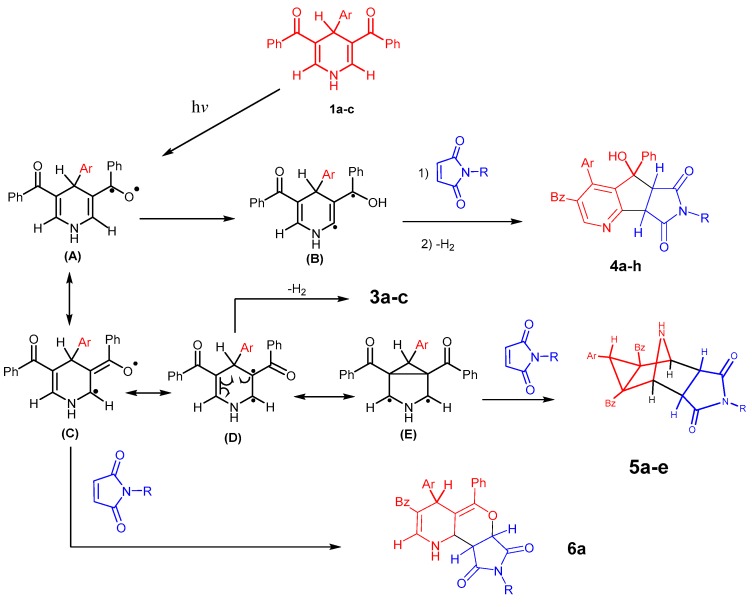
The diradical photoreaction mechanism.

The formation of the different photo-adducts **4a**–**h, 5a**–**e,** and **6a** can be satisfactorily interpreted by the formation of the diradical intermediates **A**–**D**. Diradical **A**, which is formed from the initial excitation of the (n, π*), undergoes a hydrogen shift from the β-carbon to form diradical **B**, which then undergoes cycloaddition with **2** followed by dehydrogenation to give **4**. Diradical **A** can also resonate to diradicals **C**, **D** and **E**. Trapping of the diradical **C** with **2a** yields **6a**, diradical **C** which is in resonance with **D** may also go through loss of H_2_ to give the pyridine derivatives **3a**–**c**. Finally, trapping of diradical **E** with the appropriate maleimide gives the corresponding interesting tetracyclic system **5** (Scheme 3). 

**Scheme 4:**
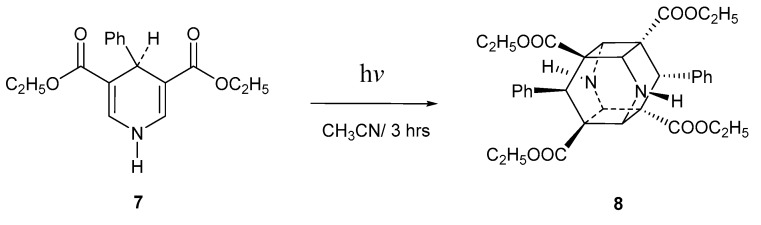
Formation of cage structure **8**.

**Figure 2:**
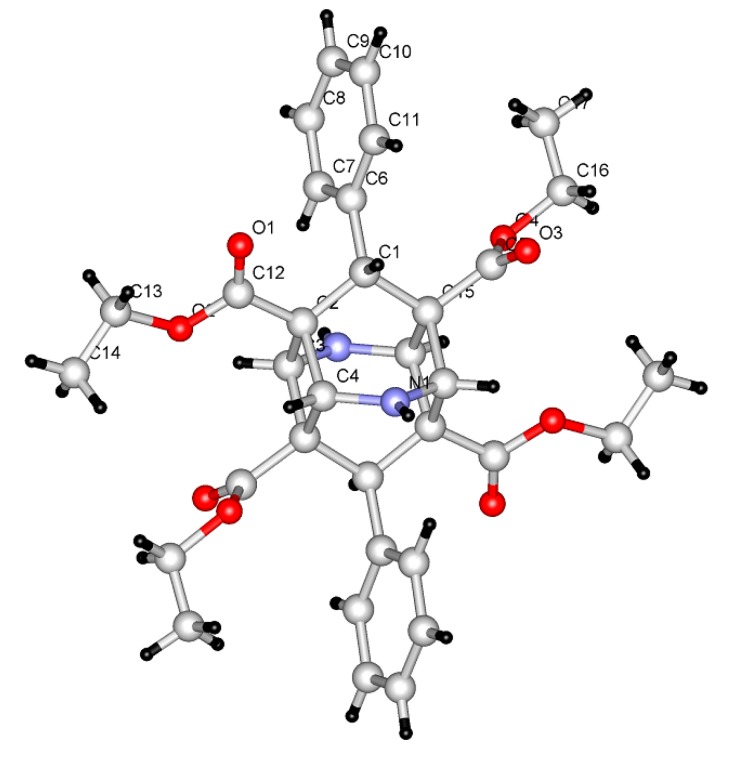
X-ray crystal structure of cage compound **8**.

Furthermore irradiation of **1a** alone in acetonitrile solution leads to the corresponding oxidized pyridine derivative **3a**. Under the same conditions irradiation of diethyl 4-phenyl-1,4-dihydropyridine-3,5-dicarboxylate (**7**) produced the cage compound **8** as shown in Scheme 4 and Figure 2. These facts showed different photochemical behavior for compounds **1a**–**c** than those reported for the other dihydropyridine derivatives. The possibility of the formation of compound **6a** by [4 + 2] thermal cycloaddition reaction has also been excluded, when attempts to react **1a** with **2a** at temperature of 180 °C for 1 h, gave only the unreacted starting material.

## 3. Experimental Section

### 3.1. General Information

Melting points were recorded on a Gallenkamp apparatus (London, United Kingdom) The UV-Vis absorption spectra were scanned by using a Cary 5 instrument (Agilent, Santa Clara, CA, USA) with dry, clean quartz cuvettes of 1.0 cm path length. IR spectra were recorded in KBr disks on JASCO FTIR 6300 spectrophotometer (JASCO, Easton, MD, USA). ^1^H-NMR (400 MHz or 600 MHz) and ^13^C-NMR (100 MHz or 150 MHz) spectra were recorded on a DPX 400 MHz NMR spectrometer (Bruker, Karlsruhe, Germany). Mass spectra were measured on (GC-MS DFS) (high resolution, high performance, tri-sector GC/MS/MS (Thermo, Bremen, Germany) and by LC-MS using LC-MS DFS (Thermo). with an API-ES/APCI ionization mode. X-Ray single crystals data were performed using a Rapid II (Rigaku, Tokyo, Japan) and X8 Prospector diffractometer (Bruker) An annular reactor model APQ40 (Applied Photo-Physics Ltd., RG 49PA, England, UK) fitted with a 400 W (λ > 290 nm) medium pressure mercury arc-lamp was used for the irradiation. The starting 1,4-dihydropyridine derivatives **1a**–**c** were synthesized as described recently [18]. 

### 3.2. General procedure for the photoreaction of 1,4-dihydropyridines ***1a**–**c*** with maleimides ***2a**–**c***

A solution of each of **1a**–**c** (1 mmol) and the appropriate **2a**–**c** (3 mmol) in acetonitrile (100 mL) in a Pyrex tube was purged with nitrogen for 20 min, and then irradiated under N_2_ atmosphere for 3 h at room temperature using a 400 W (λ > 290 nm) medium pressure mercury arc-lamp. The reaction progress was monitored by TLC. The solvent was removed under reduced pressure and the resulting residue was subject to column chromatography on silica gel (70–230 mesh) using ethyl acetate/petroleum ether (b.p. 60–80 °C) as eluent to give the corresponding photoproducts. 

*(4-Phenylpyridine-3,5-diyl)bis(phenylmethanone)* (**3a**) [18]. 

*(4-(p-Tolyl)pyridine-3,5-diyl)bis(phenylmethanone)* (**3b**) [18]. 

*(4-(4-Chlorophenyl)pyridine-3,5-diyl)bis(phenylmethanone)* (**3c**) [18]. 

*3-Benzoyl-5-hydroxy-4,5-diphenyl-5a,8a-dihydropyrrolo[3’,4’:4,5]cyclopenta[1,2-b]pyridine-6,8(5H,7H)-dione* (**4a**)*:* Colorless solid from ethyl acetate; mp. 266–268 °C; ν_max_ (KBr)/cm^−1^ 3258, 1723, 1692, 1583; δ_H_ (600 MHz, CDCl_3_) 3.6 (s, 1H), 3.9 (d, 1H, *J* 6), 4.7 (d, 1H, *J* 6), 6.8–7.5 (m, 15H), 8.1 (s, 1H), 8.8 (s, 1H); δ_C_ (150 MHz, DMSO-*d*_6_) 36.4, 47.9, 49.1, 74.2, 110.8, 115.2, 126.5, 127.2, 128.3, 128.5, 128.6, 128.8, 128.9, 129.4, 130.1, 133.6, 140.9, 146.3, 147.8, 152.1, 175.7, 176.1, 190.5; MS *m*/*z* 460.3 (M^+^, 88%), 431.1 (100%), 105.0 (65%), 77.1 (75%); HR-MS (EI) *m*/*z* [M^+^] calcd for C_29_H_20_N_2_O_4_ 460.1423, found 460.1416. 

*3-Benzoyl-5-hydroxy-7-methyl-4,5-diphenyl-5a,8a-dihydropyrrolo[3’,4’:4,5]cyclopenta[1,2-b]pyridine-6,8(5H,7H)-dione* (**4b**)*:* Colorless solid from ethyl acetate; mp. 260–262 °C; ν_max_ (KBr)/cm^−1^ 3464, 1702, 1667, 1572; δ_H_ (600 MHz, CDCl_3_) 3.1 (s, 3H), 3.7 (s, 1H), 3.9 (d, 1H, *J* 8.4), 4.7 (d, 1H, *J* 8.4), 6.8–7.5 (m, 15H), 8.8 (s, 1H); δ_C_ (150 MHz, CDCl_3_) 25.6, 51.5, 56.5, 77.23, 83.4, 123.9, 126.9, 127.4, 127.9, 128.2, 128.3, 129.6, 132.9, 133.5, 136.9, 137.2, 138.3, 146.0, 148.5, 150.6, 157.1, 173.5, 175.4, 195.7; MS *m*/*z* 474.1 (M^+^, 6%), 365.2 (83%), 288.0 (100%); HR-MS (EI) *m*/*z* [M^+^] calcd for C_30_H_22_N_2_O_4_ 474.1579, found 474.1574.

*3-Benzoyl-5-hydroxy-4,5,7-triphenyl-5a,8a-dihydropyrrolo[3’,4’:4,5]cyclopenta[1,2-b]pyridine-6,8(5H,7H)-dione* (**4c**)*:* Colorless solid from ethyl acetate; mp. 168–170 °C; ν_max_ (KBr)/cm^−1^ 3062, 1717, 1669; δ_H_ (600 MHz, CDCl_3_) 3.6 (s, 1H), 4.0 (d, 1H, *J* 8.4), 4.8 (d, 1H, *J* 8.4), 6.8–7.5 (m, 20H), 8.8 (s, 1H); δ_C_ (100 MHz, CDCl_3_) 51.5, 56.7, 83.9, 124.0, 126.5, 127.0, 127.4, 127.9, 128.2, 128.3, 128.9, 129.2, 129.7, 131.2, 131.6, 133.0, 133.5, 136.9, 137.3, 138.4, 145.7, 148.4, 150.8, 157.4, 172.4, 174.3, 195.7; MS *m*/*z* 536.1 (M^+^, 10%); HR-MS (EI) *m*/*z* [M^+^] calcd for C_35_H_24_N_2_O_4_ 536.1736, found 536.1730.

*3-Benzoyl-5-hydroxy-5-phenyl-4-(p-tolyl)-5a,8a-dihydropyrrolo[3’,4’:4,5]cyclopenta[1,2-b]pyridine-6,8(5H, 7H)dione* (**4d**)*:* Colorless solid from ethyl acetate; mp. 249–251 °C; ν_max_ (KBr)/cm^−1^ 3520, 1716, 1653; δ_H_ (400 MHz, DMSO-*d*_6_) 2.0 (s, 3H), 2.1 (s, 1H), 3.7 (d, 1H, *J* 6.2), 4.6 (d, 1H, *J* 6.2), 6.9–7.5 (m, 14H), 8.6 (s, 1H), 11.2 (s, 1H); δ_C_ (150 MHz, DMSO-*d*_6_) 20.4, 52.3, 60.5, 82.1, 93.9 125.8, 126.9, 128.3, 129.4, 130.6, 133.6, 135.1 135.5, 136.2, 138.6, 145.6, 146.5, 148.9, 158.9, 166.6, 174.7, 175.4, 186.6, 195.7; MS *m*/*z* 474.2 (M^+^, 100%), 105.0 (50%); HR-MS (EI) *m*/*z* [M^+^] calcd for C_30_H_22_N_2_O_4_ 474.1579, found 474.1574.

*3-Benzoyl-5-hydroxy-7-methyl-5-phenyl-4-(p-tolyl)-5a,8a-dihydropyrrolo[3’,4’:4,5]cyclopenta[1,2-b]pyridine-6,8(5H,7H)-dione* (**4e**)*:* Colorless solid from ethyl acetate; mp. 236–238 °C; ν_max_ (KBr)/cm^−1^ 3504, 1703, 1667; δ_H_ (400 MHz, CDCl_3_) 2.2 (s, 3H), 3.1 (s, 3H), 3.7 (s, 1H), 3.9 (d, 1H, *J* 8.4), 4.7 (d, 1H, *J* 8.4), 6.7–7.5 (m, 14H), 8.8 (s, 1H); δ_C_ (150 MHz, CDCl_3_) 21.1, 25.6, 51.4, 56.3, 83.4, 124.0, 127.4, 127.9, 128.2, 128.3, 128.7, 129.7, 130.1, 133.4, 136.9, 137.9, 138.4, 139.1, 146.3, 148.7, 150.6, 157.1, 173.57, 175.3, 195.7; MS *m*/*z* 488.4 (M^+^, 75%), 459.2 (100%), 105.0 (32%); HR-MS (EI) *m*/*z* [M^+^] calcd for C_31_H_24_N_2_O_4_ 488.1736, found 488.1731.

*3-Benzoyl-5-hydroxy-5,7-diphenyl-4-(p-tolyl)-5a,8a-dihydropyrrolo[3’,4’:4,5]cyclopenta[1,2-b]pyridine-6,8 (5H,7H)-dione* (**4f**)*:* Pale yellow solid from ethyl acetate; mp. 188–190 °C; ν_max_ (KBr)/cm^−1^ 3474, 1776, 1667; δ_H_ (600 MHz, DMSO-*d*_6_) 2.0 (s, 3H), 3.3 (s, 1H), 3.9 (d, 1H, *J* 8.4), 4.9 (d, 1H, *J* 8.4), 6.6–7.5 (m, 19H), 8.7 (s, 1H); δ_C_ (150 MHz, DMSO-*d*_6_) 21.1, 51.9, 59.9, 83.2, 125.7, 126.5, 126.9, 127.5, 127.9, 128.8, 128.9, 129.3, 129.9, 130.3, 131.1, 133.3, 134.2, 135.6, 136.9, 137.17, 139.6, 145.6, 147.3, 149.6, 159.5, 173.1, 173.7, 196.2; MS *m*/*z* 550.5 (M^+^, 100%), 521.2 (80%), 105.0 (63%); HR-MS (EI) *m*/*z* [M^+^] calcd for C_36_H_26_N_2_O_4_ 550.1892, found 550.1888.

*3-Benzoyl-4-(4-chlorophenyl)-5-hydroxy-7-methyl-5-phenyl-5a,8a-dihydropyrrolo[3’,4’:4,5]cyclopenta[1,2-b]pyridine-6,8(5H,7H)-dione* (**4g**)*:* Colorless solid from ethyl acetate; mp. 196–198 °C; ν_max_ (KBr)/cm^−1^ 3383, 1712, 1688; δ_H_ (400 MHz, CDCl_3_) 3.1 (s, 3H), 3.8 (s, 1H), 3.9 (d, 1H, J 8.4), 4.7 (d, 1H, J 8.4), 6.8–7.5 (m, 14H), 8.8 (s, 1H); δ_C_ (100 MHz, CDCl_3_) 25.6, 51.5, 56.3, 78.6, 83.2, 123.9, 127.1, 127.5, 128.4, 129.6, 131.4, 133.7, 134.1, 135.2, 136.7, 137.0, 138.4, 145.7, 147.3, 150.7, 157.1, 173.4, 175.6, 195.4; MS *m*/*z* 508.1 (M^+^, 3%), 510.2 (M^+^+2, 5%), 288.2 (100%), 105.0 (56%); HR-MS (EI) *m*/*z* [M^+^] calcd for C_30_H_21_ClN_2_O_4_ 508.1189, found 508.1184.

*3-Benzoyl-4-(4-chlorophenyl)-5-hydroxy-5,7-diphenyl-5a,8a-dihydropyrrolo[3’,4’:4,5]cyclopenta[1,2-b]pyridine-6,8(5H,7H)-dione* (**4h**)*:* Colorless solid from ethyl acetate; mp. 178–180 °C; ν_max_ (KBr)/cm^−1^ 3561, 1711, 1668; δ_H_ (400 MHz, DMSO-*d*_6_) 3.3 (s, 1H), 3.9 (d, 1H, J 8.8), 4.8 (d, 1H, J 8.8), 6.9–7.5 (m, 19H), 8.7 (s, 1H); δ_C_ (150 MHz, DMSO-*d*_6_) 52.0, 59.9, 83.1, 125.9, 126.8, 127.1, 127.7, 127.9, 128.9, 129.1, 129.4, 130.1, 131.4, 132.9, 133.3, 134.5, 136.6, 137.1, 137.6, 139.7, 145.5, 145.9, 150.0, 159.9, 173.1, 173.7, 196.0; MS *m*/*z* 570.2 (M^+^, 93%), 572.2 (M+2, 36%), 542.2 (95%), 105.0 (100%); HR-MS (EI) *m*/*z* [M^+^] calcd for C_35_H_23_ClN_2_O_4_ 570.1346, found 570.1341.

*8,10-Dibenzoyl-9-phenyl-4,11-diazatetracyclo[5,3,1,0^2,6^,0^8,10^]undecane-3,5-dione* (**5a**)*:* Colorless solid from ethyl acetate; mp. 275–277 °C; ν_max_ (KBr)/cm^−1^ 3469, 3367, 1773, 1708; δ_H_ (600 MHz, CDCl_3_) 3.6 (s, 2H), 3.7 (s, 1H), 4.7 (s, 2H), 7.3 (s, 1H), 7.4–7.9 (m, 15H), 7.9 (t, 1H); δ_C_ (150 MHz, DMSO-*d*_6_) 39.7, 48.8, 51.3, 62.5, 127.5, 128.1, 128.4, 128.8, 130.3, 132.9, 134.9, 138.6, 177.0, 198.1; MS *m*/*z* 462.20 (M^+^, 11%), 288.1 (66%), 105.0 (100%); HR-MS (EI) *m*/*z* [M^+^] calcd for C_29_H_22_N_2_O_4_ 462.1579, found 462.1574.

*8,10-Dibenzoyl-4-methyl-9-phenyl-4,11-diazatetracyclo[5,3,1,0^2,6^,0^8,10^]undecane-3,5-dione* (**5b**)*:* Colorless solid from ethyl acetate; mp. 248–250 °C; ν_max_ (KBr)/cm^−1^ 3411, 1753, 1718; δ_H_ (400 MHz, CDCl_3_) 2.8 (s, 3H), 3.5 (d, 2H, *J* 2.4), 3.7 (s, 1H), 4.5 (d, 2H, *J* 1.2), 7.2–7.6 (m, 15H), 7.9 (t, 1H); δ_C_ (150 MHz, CDCl_3_) 23.9, 38.8, 46.6, 50.6, 61.6, 126.6, 127.2, 127.5, 127.9, 129.4, 131.9, 134.1, 137.8, 176.4, 197.2; MS *m*/*z* 476.4 (M^+^, 2%), 288.2 (12%), 105.0 (100%); HR-MS (EI) *m*/*z* [M^+^] calcd for C_30_H_24_N_2_O_4_ 476.1736, found 476.1346.

*8,10-Dibenzoyl-4-methyl-9-p-tolyl-4,11-diazatetracyclo[5,3,1,0^2,6^,0^8,10^]undecane-3,5-dione* (**5c**)*:* Colorless solid from ethyl acetate; mp. 268–270 °C; ν_max_ (KBr)/cm^−1^ 3342, 1773, 1701; δ_H_ (400 MHz, CDCl_3_) 2.4 (s, 3H), 2.8 (s, 3H), 3.5 (s, 2H), 3.6 (s, 1H), 4.5 (s, 2H), 7.3–7.6 (m, 14H), 7.9 (t, 1H); δ_C_ (100 MHz, CDCl_3_) 21.1, 24.7, 39.6, 47.5, 51.4, 62.5, 127.3, 128.1, 128.8, 130.9, 131.6, 132.9, 138.5, 138.6, 177.4, 198.2; MS *m*/*z* 490.5 (M^+^, 8%), 288.5 (28%), 105.0 (100%); HR-MS (EI) *m*/*z* [M^+^] calcd for C_31_H_26_N_2_O_4_ 490.1892, found 490.1888.

*9-p-Chlorophenyl-8,10-dibenzoyl-4,11-diazatetracyclo[5,3,1,0^2,6^,0^8,10^]undecane-3,5-dione* (**5d**)*:* Colorless solid from ethyl acetate; mp. 268–270 °C; ν_max_ (KBr)/cm^−1^ 3399, 1741, 1726; δ_H_ (600 MHz, CDCl_3_) 3.6 (s, 2H), 3.6 (s, 1H), 4.6 (s, 2H), 7.3–7.6 (m, 15H), 7.9 (t, 1H); δ_C_ (150 MHz, CDCl_3_) 29.9, 39.0, 51.6, 62.6, 128.2, 129.1, 129.1, 130.8, 133.2, 133.7, 134.7, 138.7, 176.9, 197.9; MS *m*/*z* 496.3 (M^+^,12%), 294.2, (84%), 105.0 (100%); HR-MS (EI) *m*/*z* [M^+^] calcd for C_29_H_21_ClN_2_O_4_ 496.1189, found 496.1184.

*9-p-Chlorophenyl-8,10-dibenzoyl-4-methyl-4,11-diazatetracyclo[5,3,1,0^2,6^,0^8,10^]undecane-3,5-dione* (**5e**)*:* Colorless solid from ethyl acetate; mp. 296–298 °C; ν_max_ (KBr)/cm^−1^ 3330, 1777, 1703; δ_H_ (600 MHz, CDCl_3_) 2.8 (s, 3H), 3.5 (s, 2H), 3.6 (s, 1H), 4.5 (s, 2H), 7.3–7.6 (m, 14H), 7.9 (t, 1H); δ_C_ (150 MHz, CDCl_3_) 24.7, 38.8, 51.6, 62.4, 128.0, 128.8, 128.8, 130.5, 132.9, 133.5, 134.4, 138.6, 177.2, 197.7; MS *m*/*z* 510.2 (M^+^, 10%), 513.2 (M^+^+2, 6.2%), 105.0 (100%); HR-MS (EI) *m*/*z* [M^+^] calcd for C_30_H_23_ClN_2_O_4_ 510.1346, found 510.1340.

*3-Benzoyl-4,5-diphenyl-1,6a,9a,9b-tetrahydropyrrolo[3’,4’:5,6]pyrano[4,3-b]pyridine-7,9(4H,8H)-dione* (**6a**)*:* Colorless solid from ethyl acetate; mp. 223–225 °C; ν_max_ (KBr)/cm^−1^ 3334, 1721; δ_H_ (600 MHz, DMSO-*d*_6_) 3.9 (t, 1H, *J* 7.8 ), 4.8 (d, 1H, *J* 8.4), 4.9 (s, 1H), 5.2 (d, 1H, *J* 8.4), 7.17 (s, 1H), 7.2–7.4 (m, 15H), 7.4 (s, 1H), 11.7 (s, 1H); δ_C_ (150 MHz, DMSO-*d*_6_) 36.4, 47.9, 49.1, 74.3, 110.8, 115.2, 126.5, 127.2, 128.3, 128.5, 128.6, 128.7, 128.9, 129.4, 130.1, 133.9, 140.9, 146.3, 147.8, 152.1, 175.7, 176.1, 190.5; MS *m*/*z* 462.1 (M^+^, 6%), 365.2 (85%), 288.0 (100%); HR-MS (EI) *m*/*z* [M^+^] calcd for C_29_H_22_N_2_O_4_ 462.1579, found 462.1574.

## 4. Conclusions

The present investigation presents a novel photochemical behavior of 4-aryl-3,5-dibenzoyl-1,4-dihydropyridine derivatives **1a**–**c** with maleimides **2a**–**c** using medium pressure Hg-arc lamp at λ > 290 nm and afforded three different cycloadducts **4**, **5**, **6**. These results indicate that compounds **1** undergo intermolecular cycloaddition reactions through three biradical intermediates and behave photochemically different than those reported previously for the analogous 3,5-diacetyl and 3,5-dicarboxylic acid derivatives. The present work also offers interesting simple access to novel tricyclic and tetracyclic nitrogen heterocyclic ring systems of potential biological and synthetic applications.

## Figures and Tables

**Table 1:** Photoproducts from irradiation of 1,4-DHPs **1a**–**c** with maleimides **2a**–**c.**

Entry *	Reactants		Photoproducts (Yield %)			
	1a–c	2a–c	3a–c	4a–h	5a–e	6a
1	**1a**	**2a**	**3a** (48)	**4a** (23)	**5a** (15)	**6a** (13)
2	**1b**	**2a**	**3b** (45)	**4b** (28)	**5b** (12)	-
3	**1c**	**2a**	**3c** (44)	**4c** (29)	-	-
4	**1a**	**2b**	**3a** (41)	**4d** (32)	-	-
5	**1b**	**2b**	**3b** (41)	**4e** (27)	**5c** (13)	-
6	**1c**	**2b**	**3c** (39)	**4f** (34)	-	-
7	**1a**	**2c**	**3a** (37)	-	**5d** (23)	-
8	**1b**	**2c**	**3b** (39)	**4g** (29)	**5e** (10)	-
9	**1c**	**2c**	**3c** (42)	**4h** (27)	-	-

* Reaction conditions: a solution of **1a**–**c** (1 mmol) with **2a**–**c** (3 mmol) was irradiated at λ > 290 nm in acetonitrile (100 mL) for 3 h under N_2_.

## Supplementary Materials

Crystallographic data of (excluding structure factors) for the structure in this paper have been deposited with the Cambridge Crystallographic Data Centre as supplementary publication nos. CCDC 1475111 (**5a**), CCDC 1475112 (**5b**), CCDC 1475113 (**6a**), CCDC 1475114 (**4d**) and CCDC 1475115 (**8**). Copies of the data can be obtained, free of charge, on application to CCDC, 12 Union Road, Cambridge CB2 1EZ, UK, (fax: +44-(0)1223-336033 or e-mail: deposit@ccdc.cam.ac.uk. Supplementary materials can be accessed at: http://www.mdpi.com/1420-3049/21/7/866/s1.
