# Supplementary material for: Photochemistry of 1,4-Dihydropyridine Derivatives: Diradical Formation, Delocalization and Trapping as a Route to Novel Tricyclic and Tetracyclic Nitrogen Heterocyclic Ring Systems
Source: Molecules. 2016 Jun 30;21(7):866. doi: 10.3390/molecules21070866 (PMC6274155; doi:10.3390/molecules21070866)
Supplement: Supplementary file 1 [file molecules-21-00866-s001.pdf]

Supplementary Materials: Photochemistry of 1,4-dihydropyridine Derivatives: Diradical Formation, Delocalization and Trapping as a Route to Novel Tricyclic and Tetracyclic Nitrogen Heterocyclic Ring Systems

Nader A. Al-Jalal, Yehia A. Ibrahim, Nouria A. Al-Awadi, Maher R. Ibrahim and Osama M. Sayed

Table S1. Optimization of photoreaction conditions of **1a** with **2a**.

Run	Lamp/time	Vessel	Solvent	Molar ratio of 1a:2a	Photoproducts%			
					3a	4a	5a	6a
1	16 W/6 h	Quartz	CH_3CN	1:1	40	-	-	-
2	16 W/16 h	Quartz	CH_3CN	1:1	45	-	-	-
3	16 W/24 h	Quartz	CH_3CN	1:1	65	-	-	-
4	400 W/3 h	Quartz	$CHCl_3$	1:2	70	11	7.5	-
5	400 W/3 h	Pyrex	$CH_3C_6H_5$	1:2	-	-	-	-
6	400 W/3 h	Pyrex	CH_2Cl_2	1:2	40	12	8	-
7	400 W/3 h	Pyrex	CH_3CN	1:3	48	23	15	13

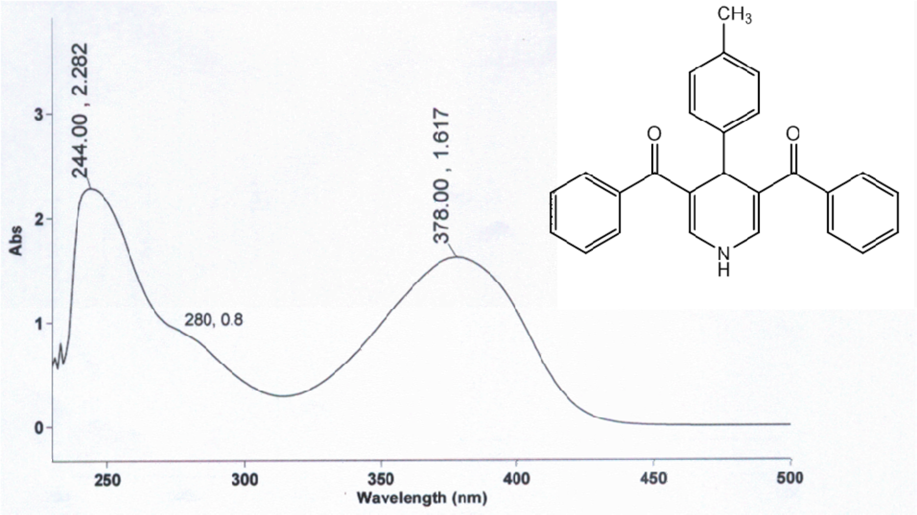

Figure S1. UV-spectrum of 1,4-DHP **3b**.

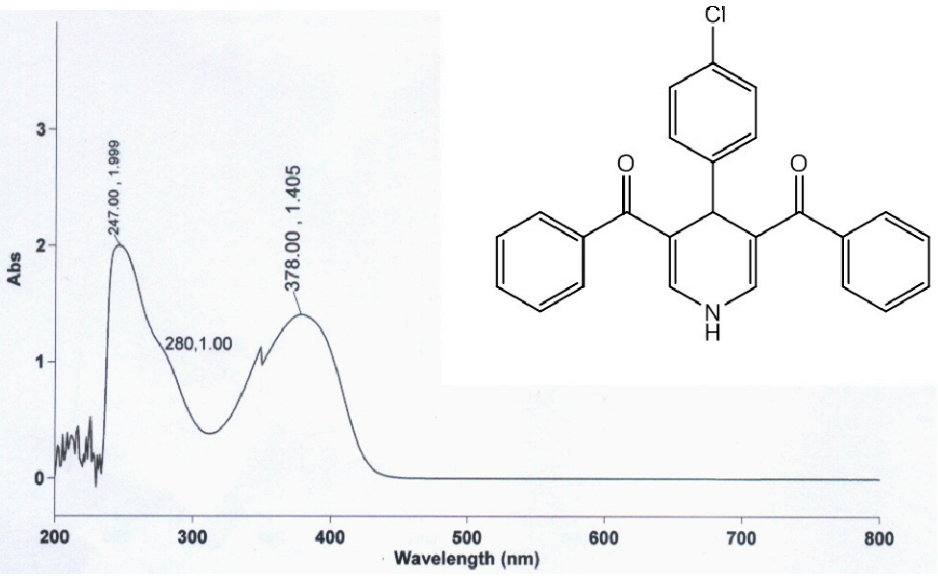

Figure S2. UV-spectrum of 1,4-DHP **3c**.

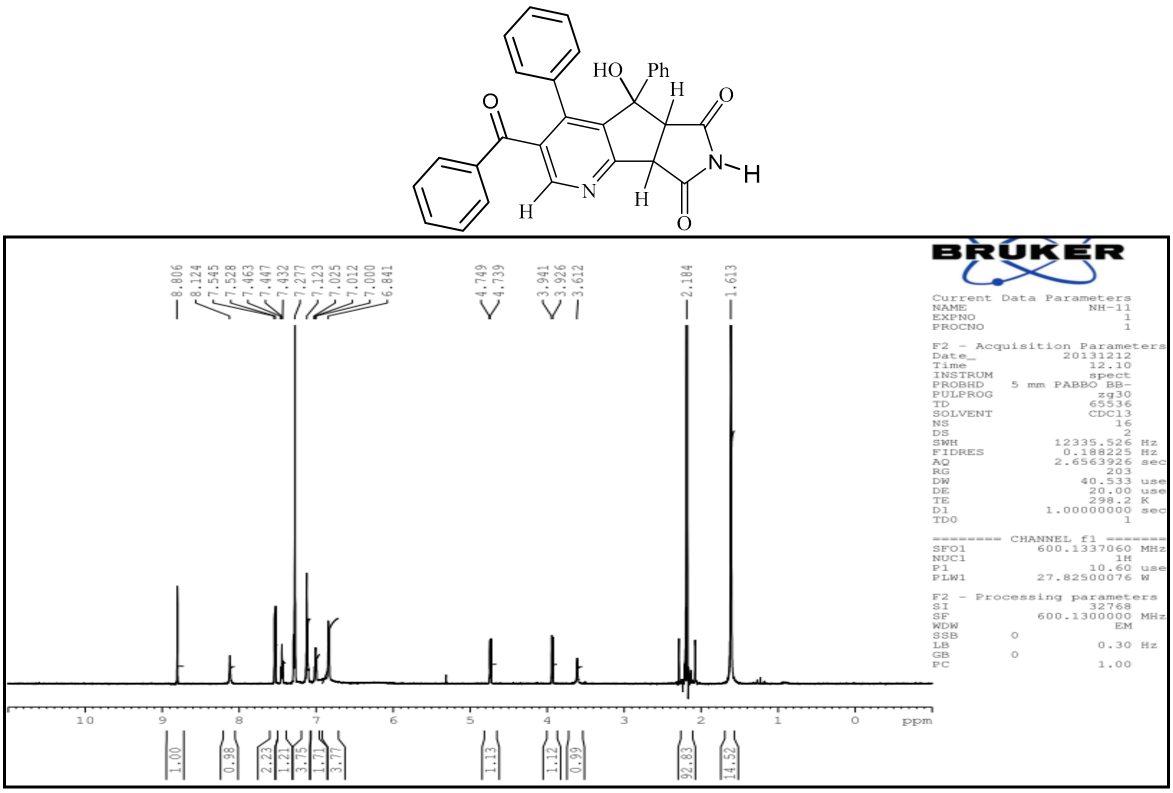

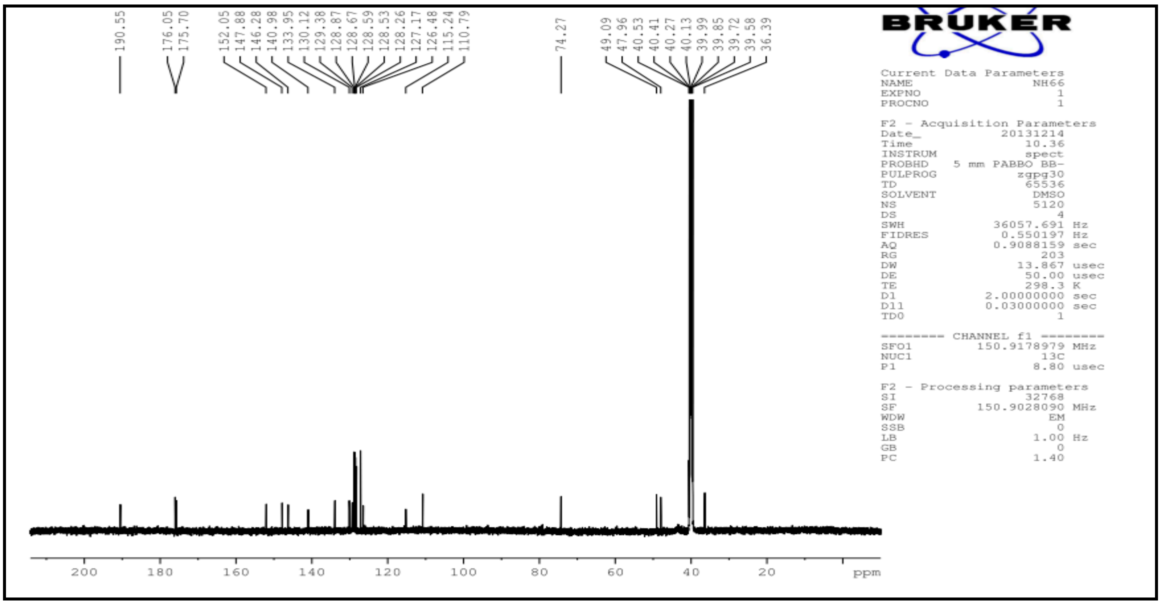

Figure S3. ^{1}H-NMR and ^{13}C-NMR spectra of compound **4a**.

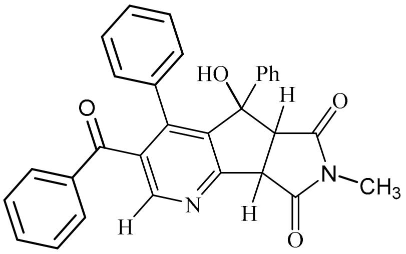

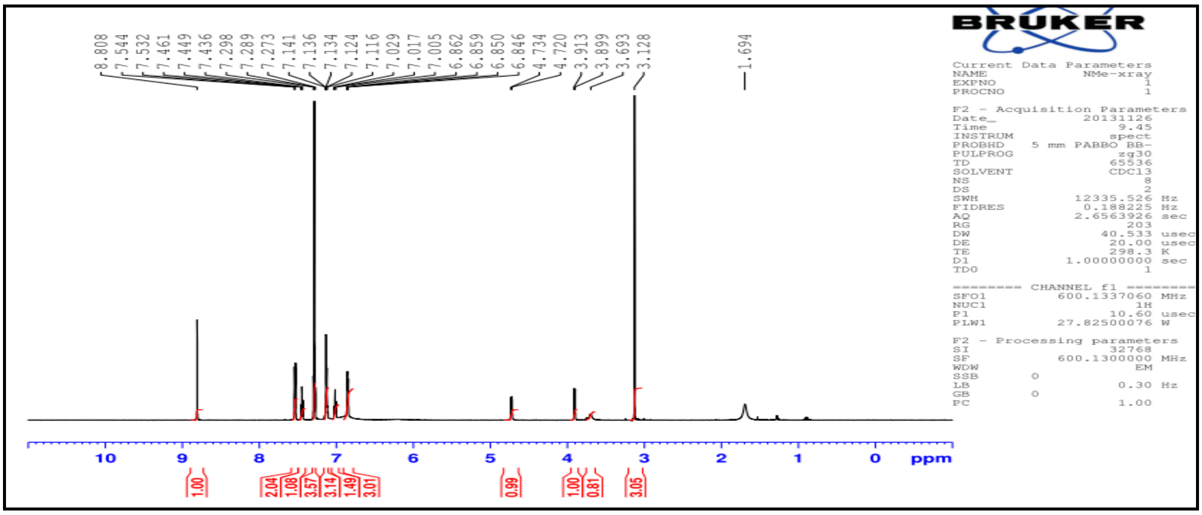

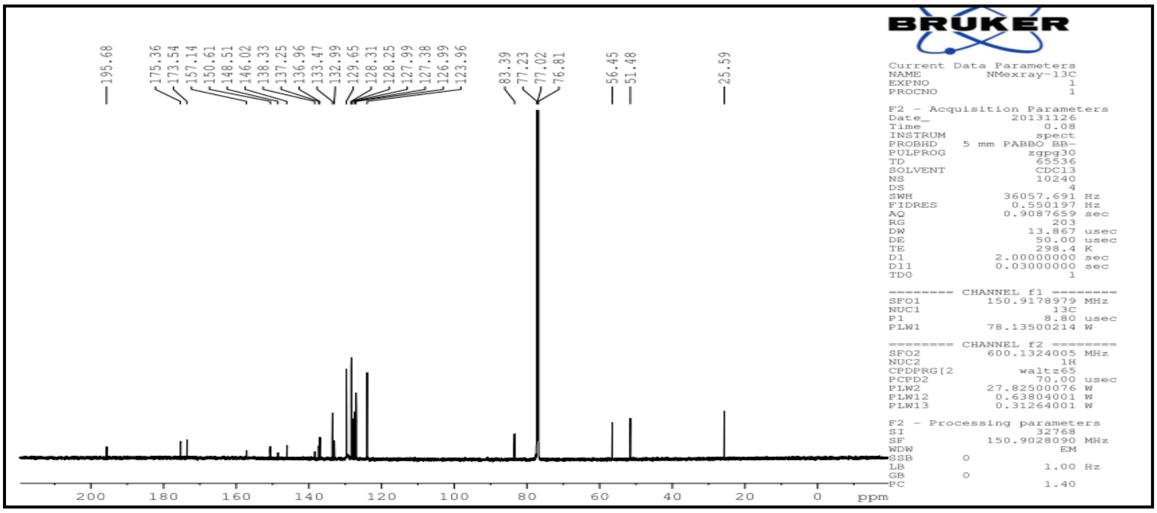

Figure S4. ^{1}H-NMR and ^{13}C-NMR spectra of compound **4b**.

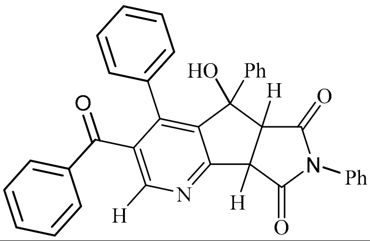

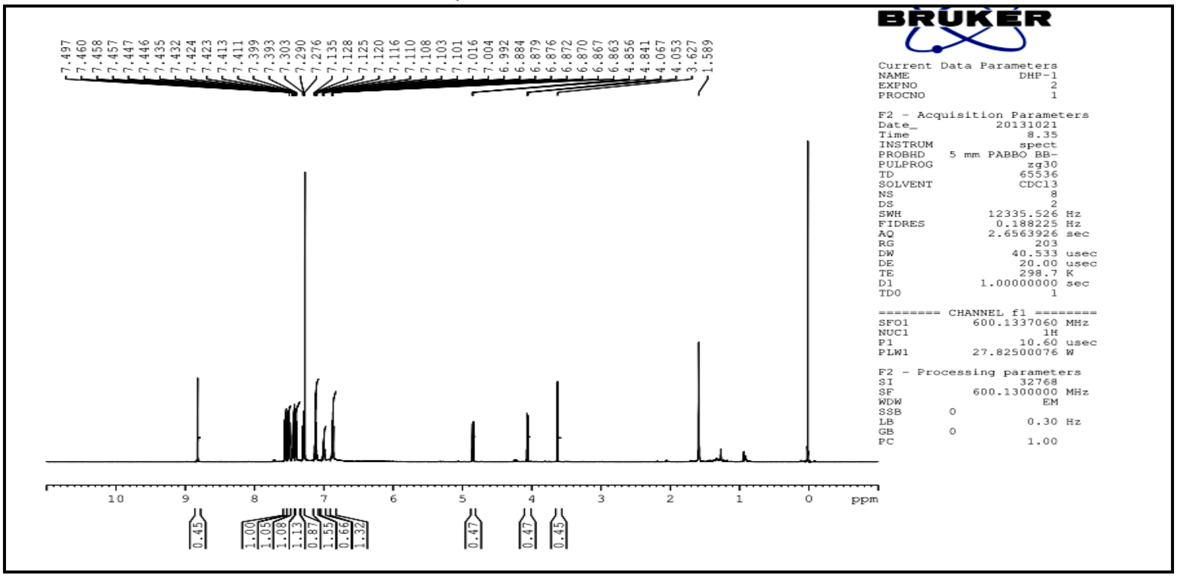

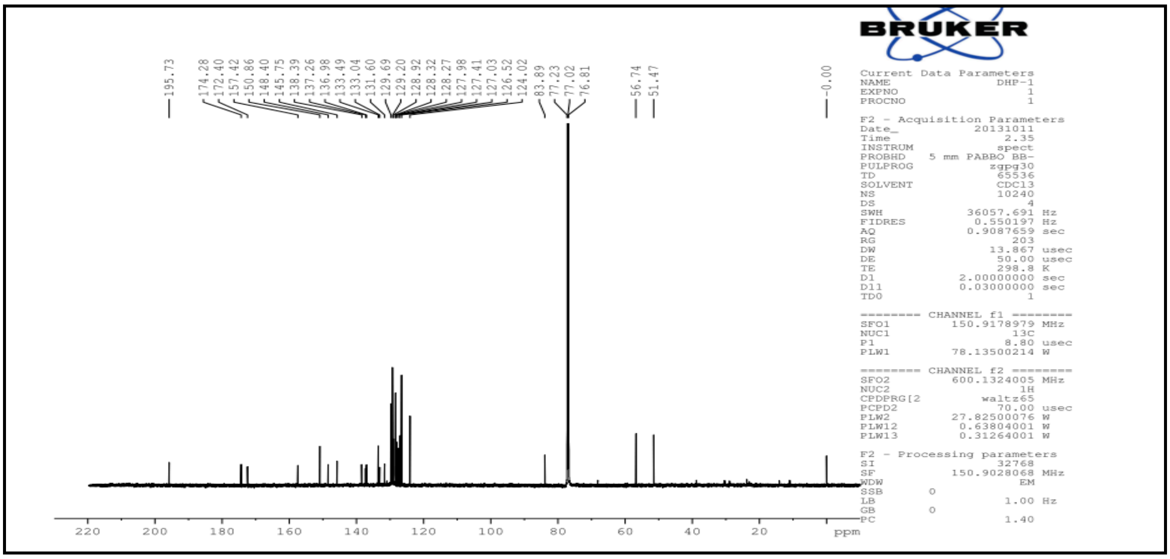

Figure S5. ^{1}H-NMR and ^{13}C-NMR spectra of compound **4c**.

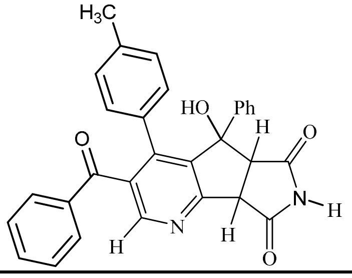

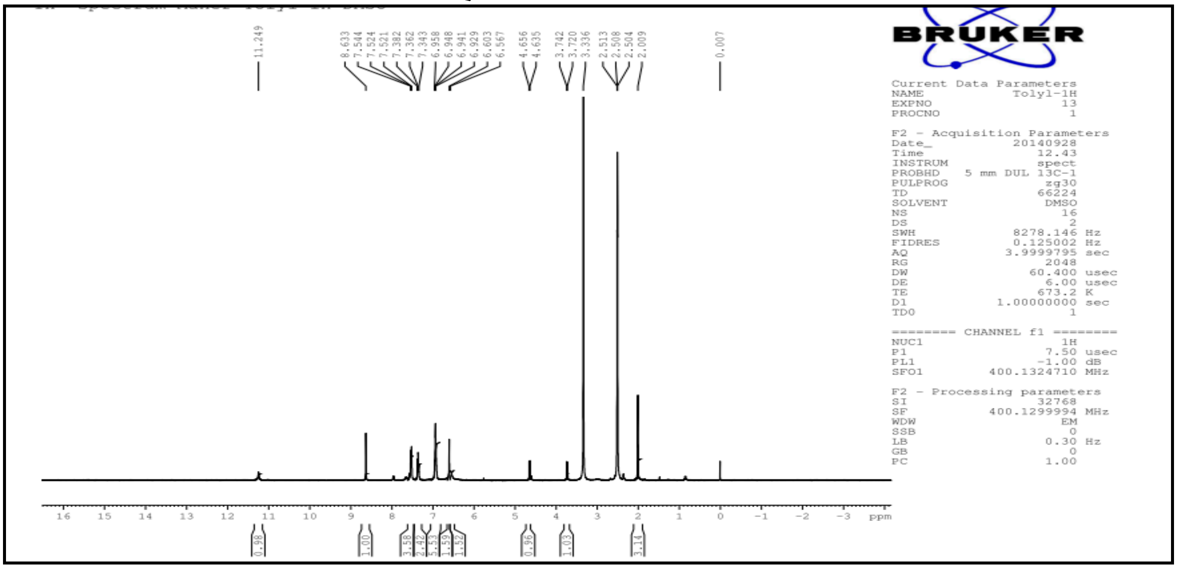

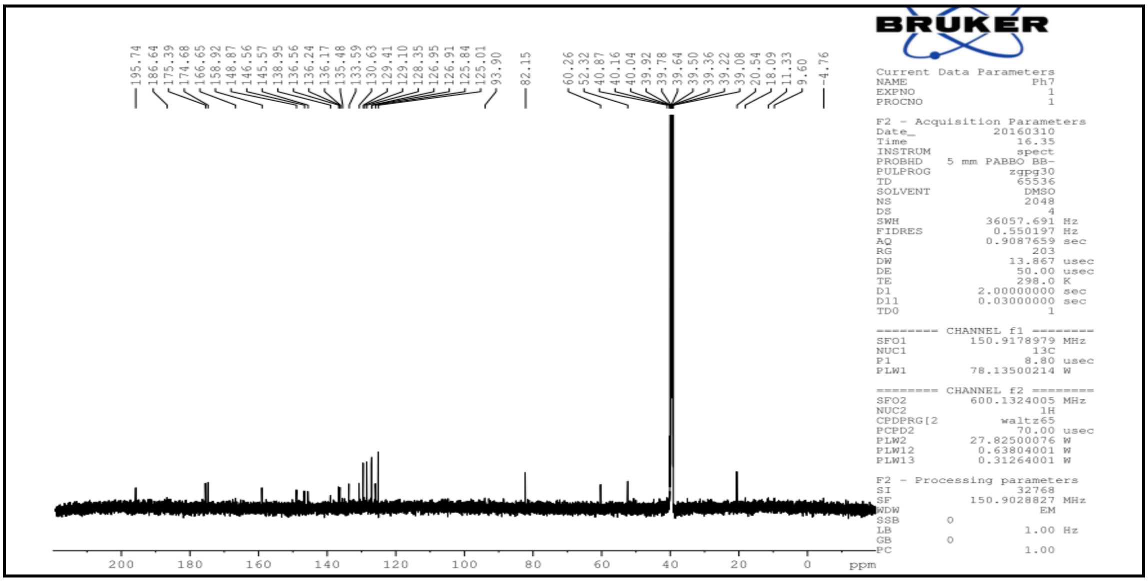

Figure S6. ^{1}H-NMR and ^{13}C-NMR spectra of compound **4d**.

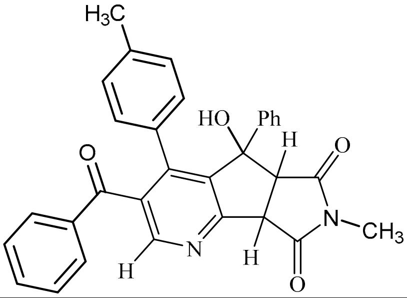

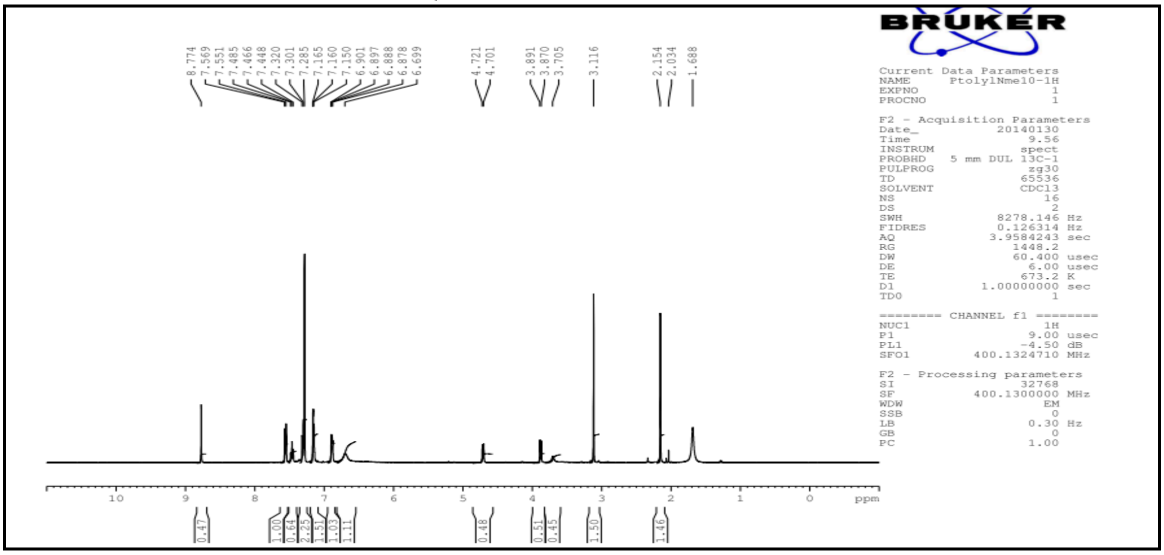

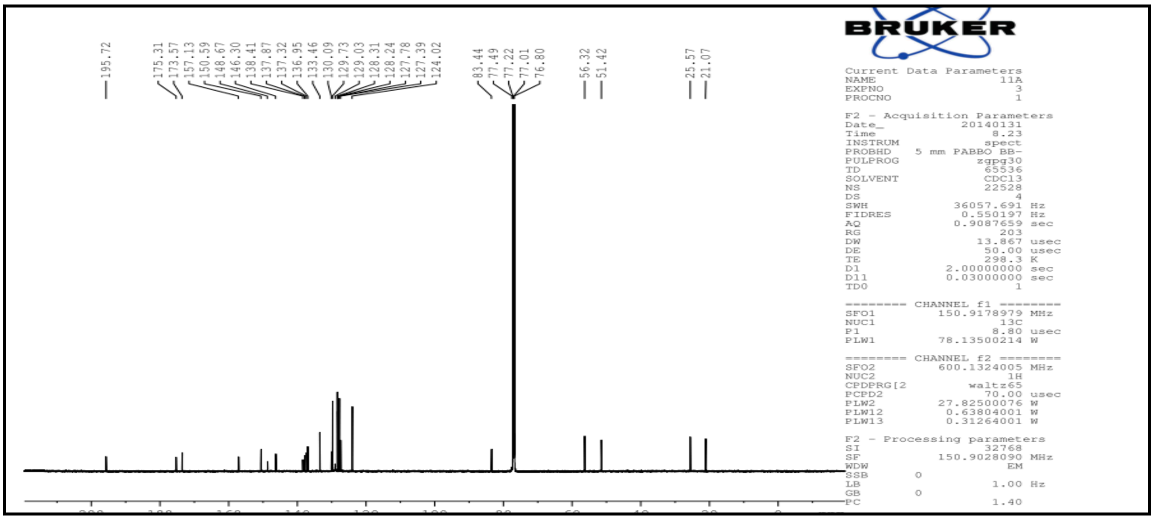

Figure S7. ^{1}H-NMR and ^{13}C-NMR spectra of compound **4e**.

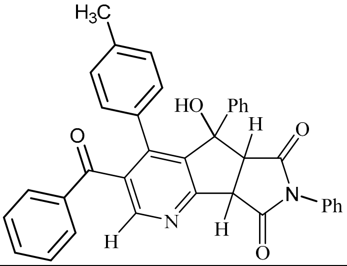

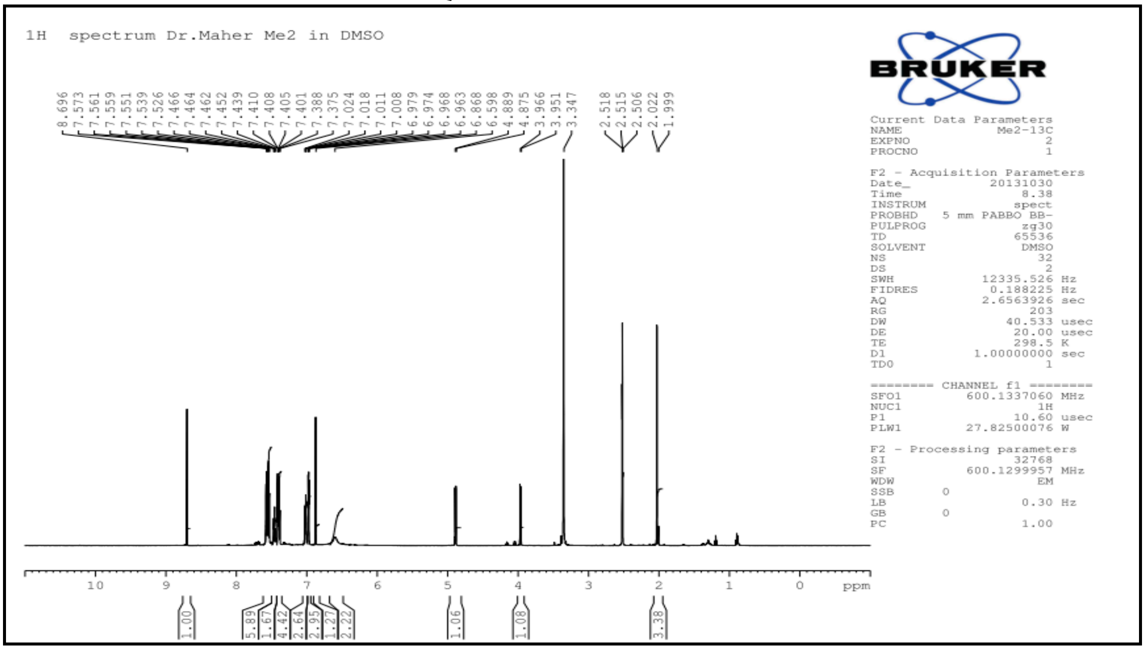

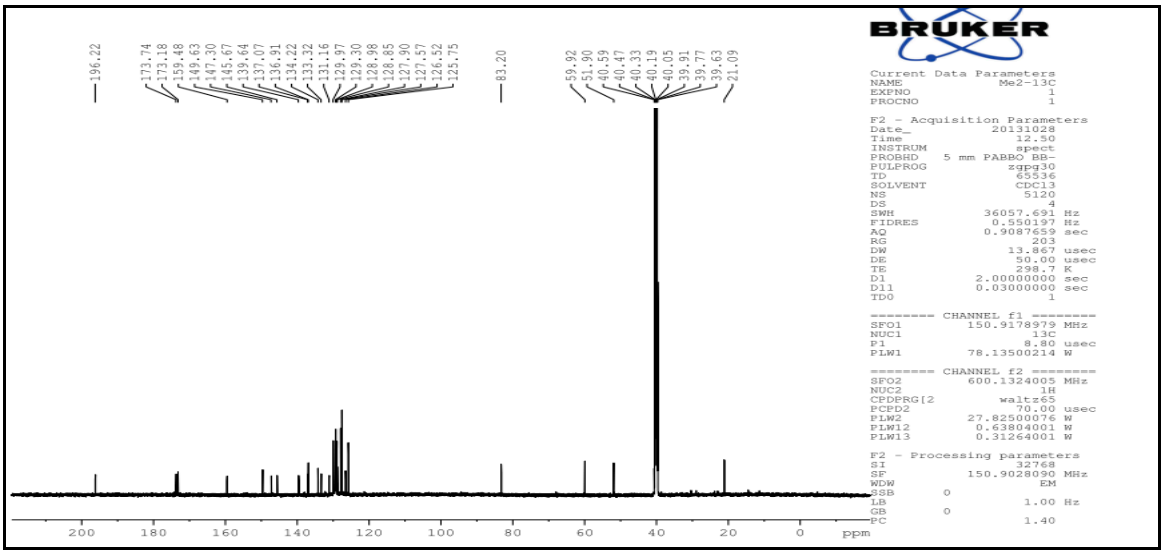

Figure S8. ^{1}H-NMR and ^{13}C-NMR spectra of compound **4f**.

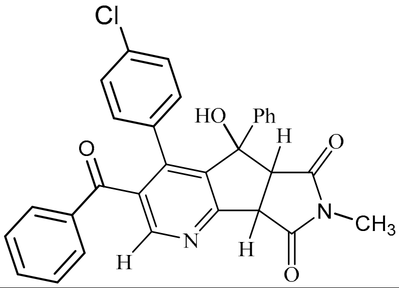

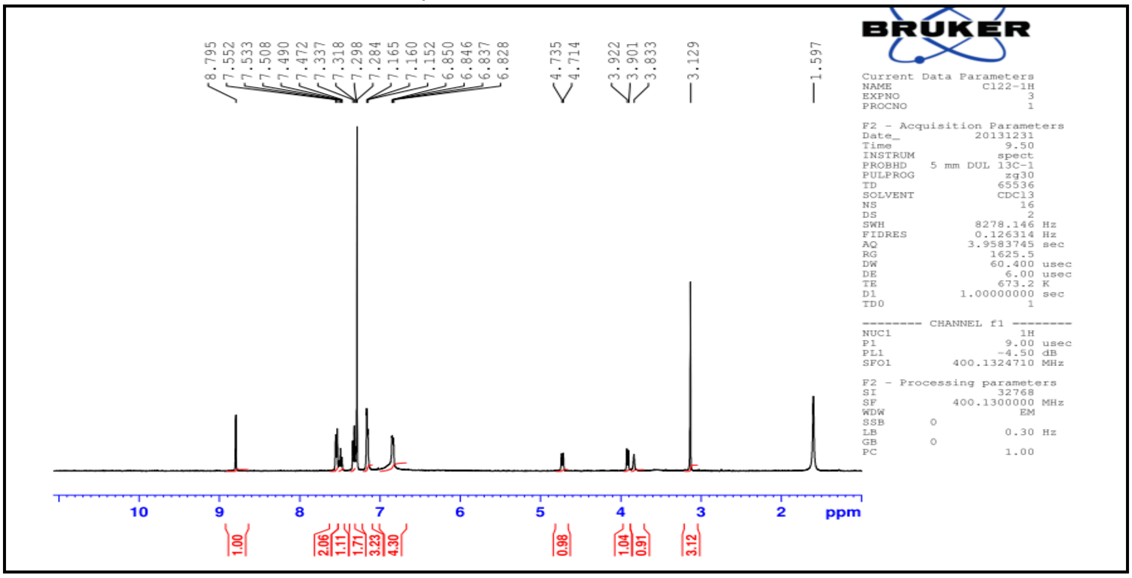

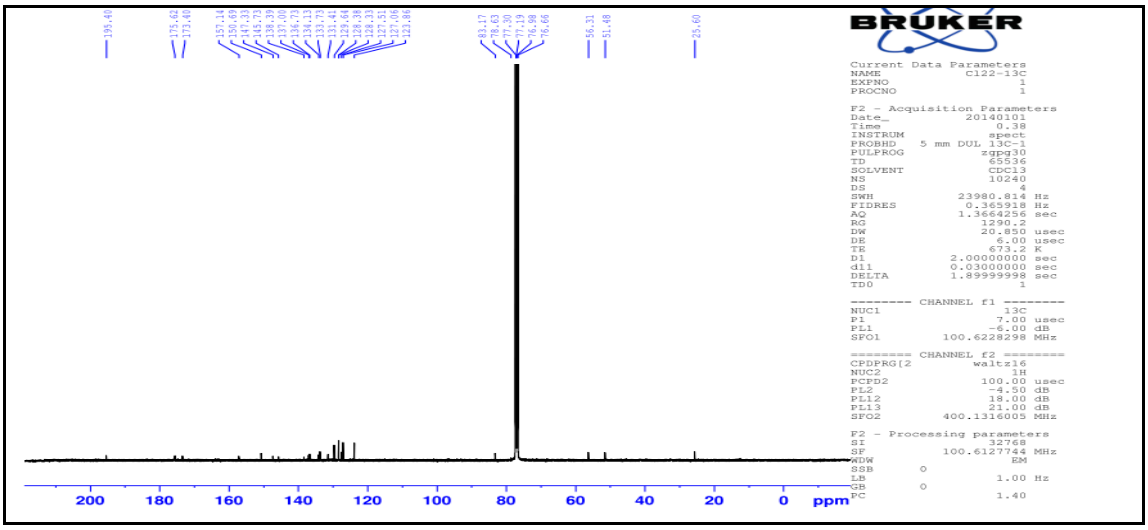

Figure S9. ^{1}H-NMR and ^{13}C-NMR spectra of compound **4g**.

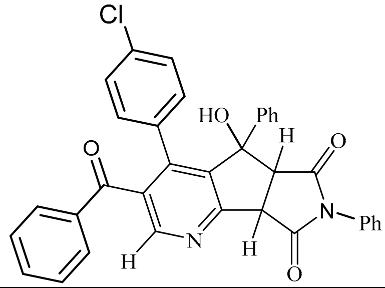

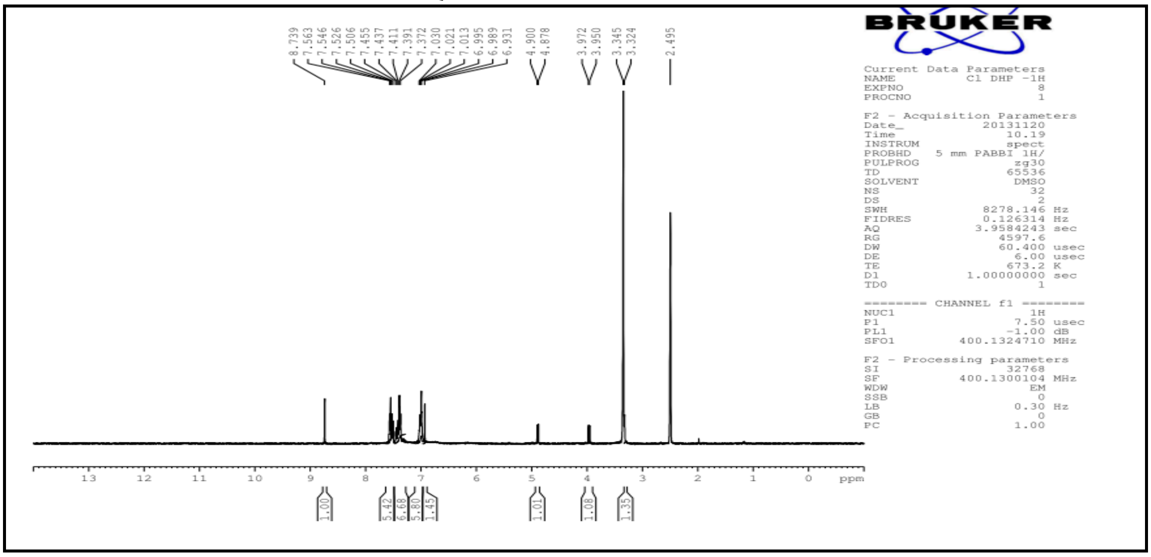

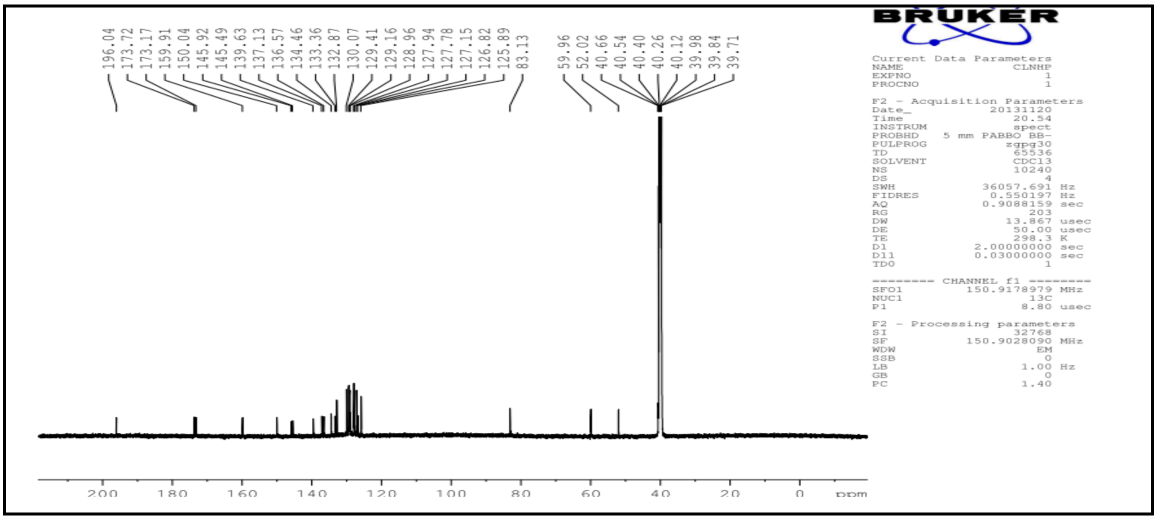

Figure S10. ^{1}H-NMR and ^{13}C-NMR spectra of compound **4h**.

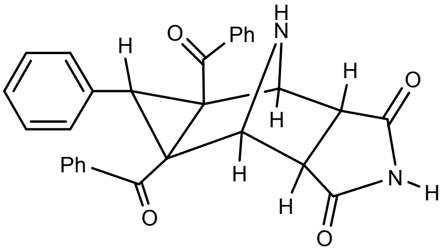

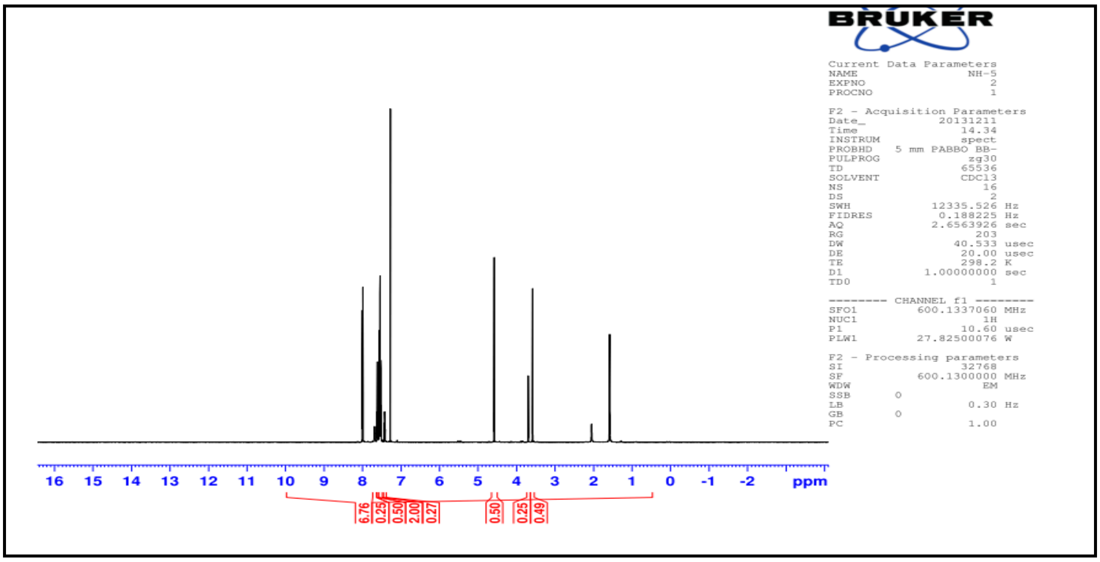

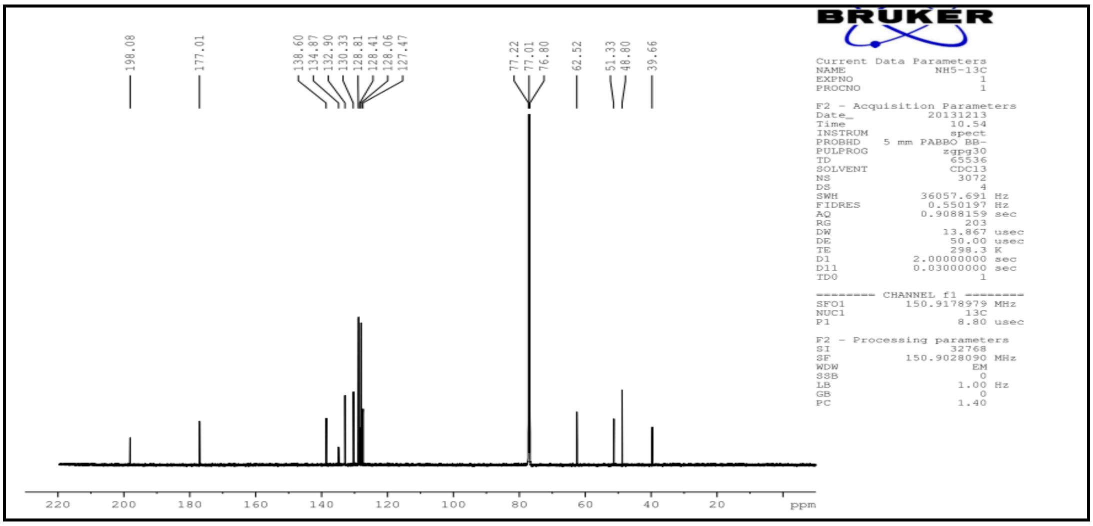

Figure S11. ^{1}H-NMR and ^{13}C-NMR spectra of compound **5a**.

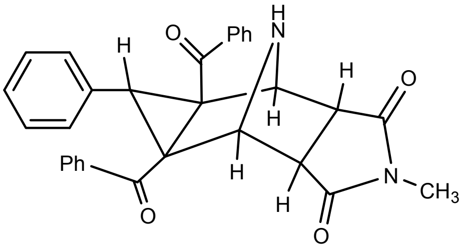

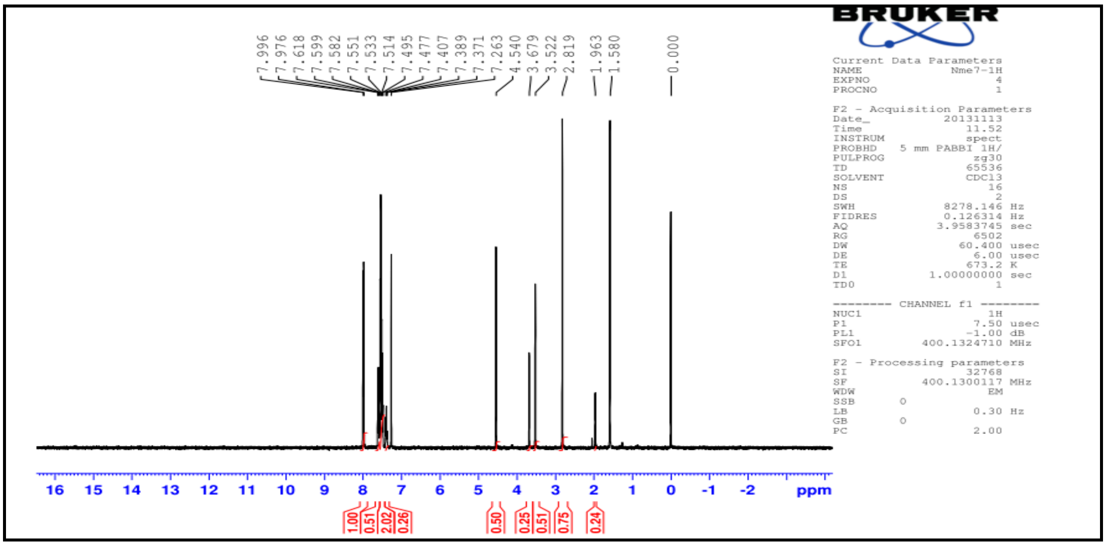

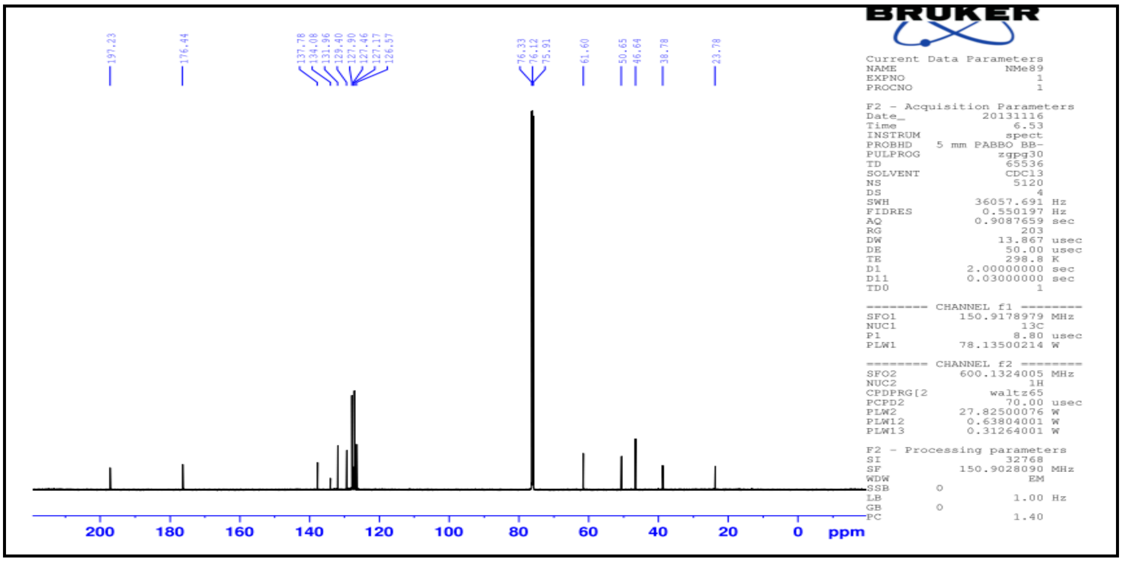

Figure S12. ^{1}H-NMR and ^{13}C-NMR spectra of compound **5b**.

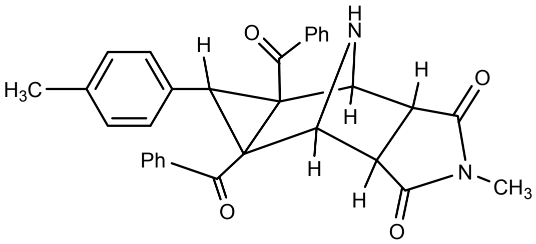

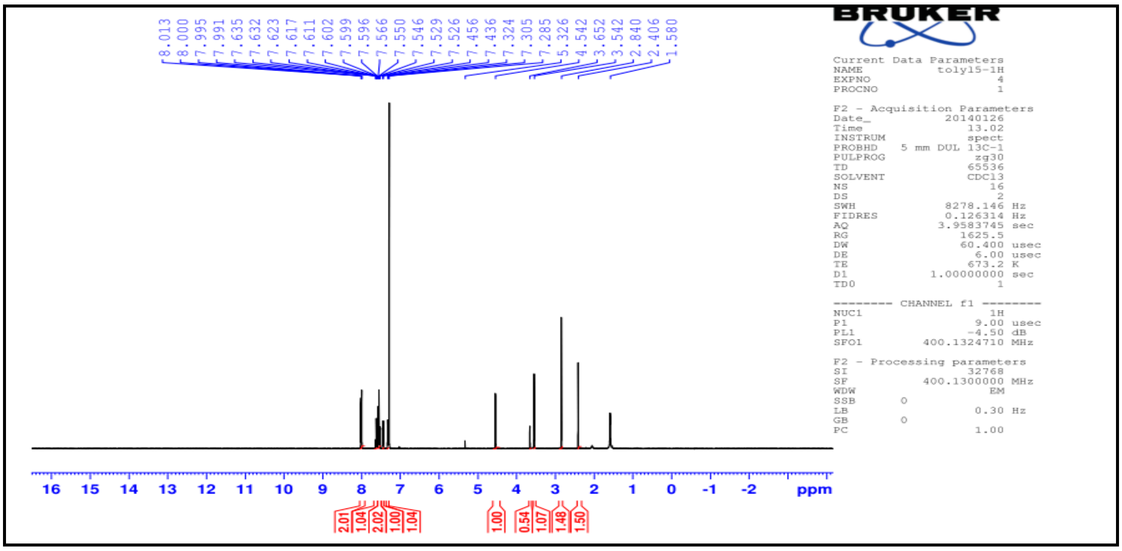

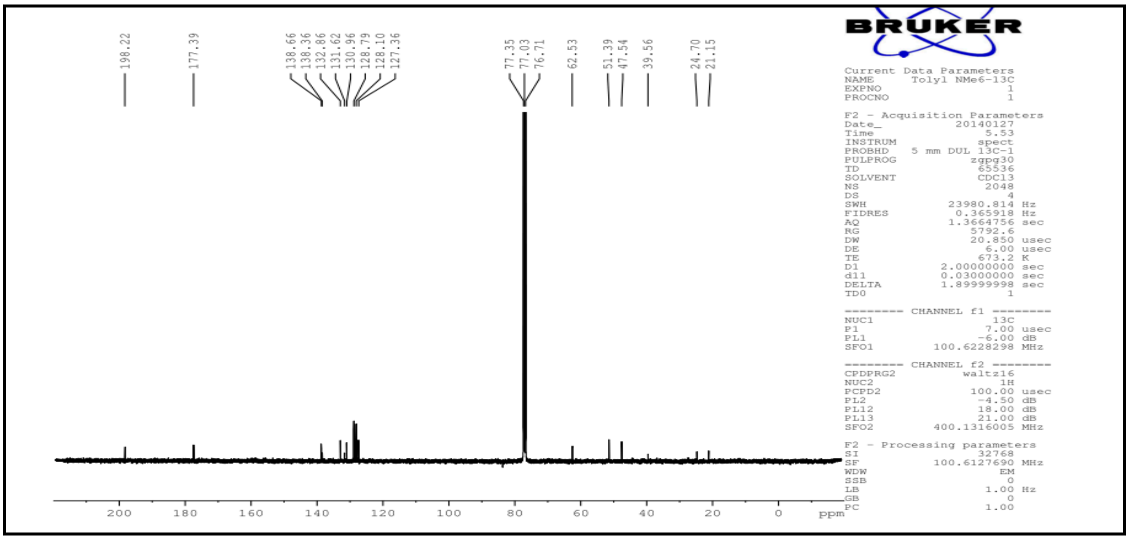

Figure S13. ^{1}H-NMR and ^{13}C-NMR spectra of compound **5c**.

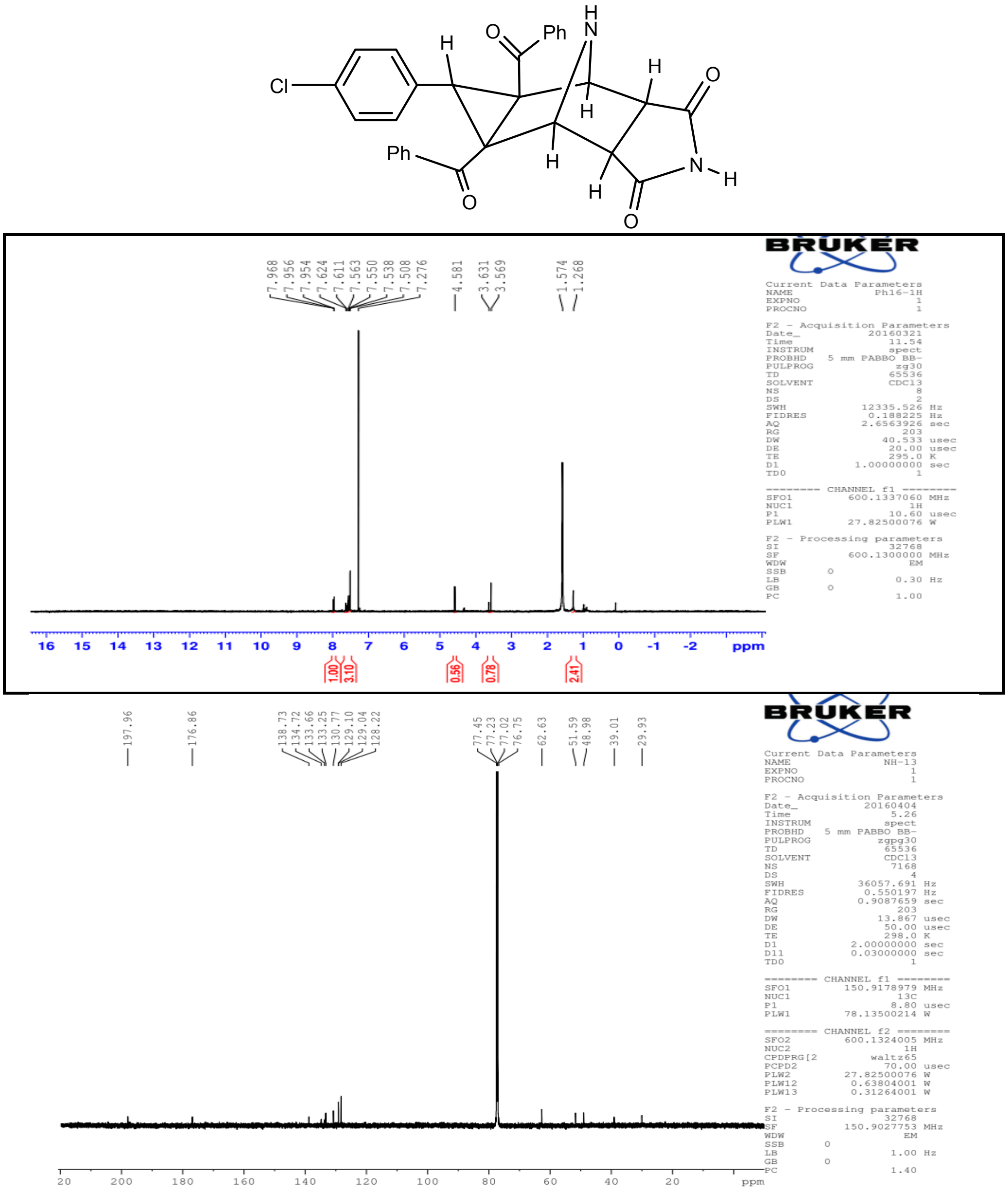

Figure S14. ^{1}H-NMR and ^{13}C-NMR spectra of compound **5d**.

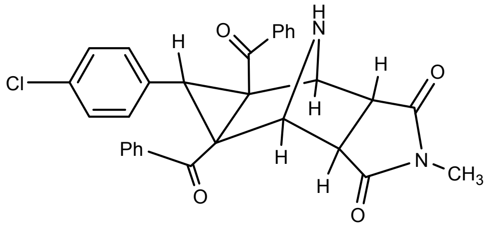

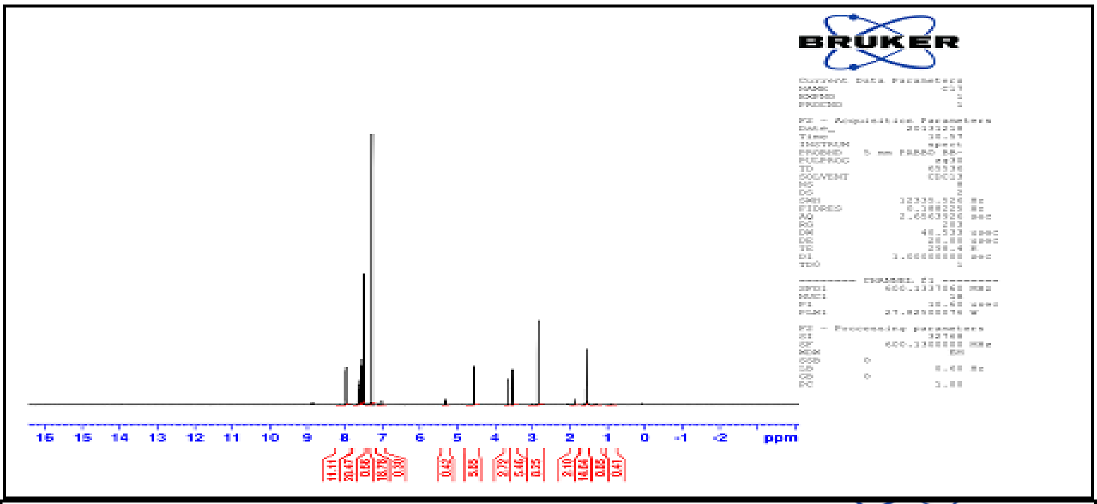

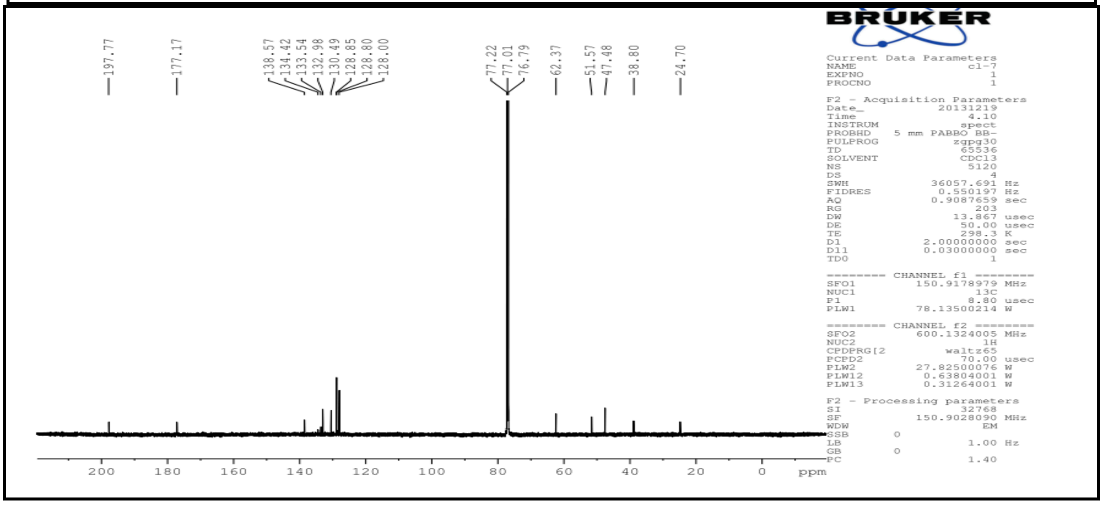

Figure S15. ^{1}H-NMR and ^{13}C-NMR spectra of compound **5e**.

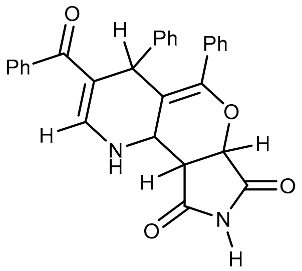

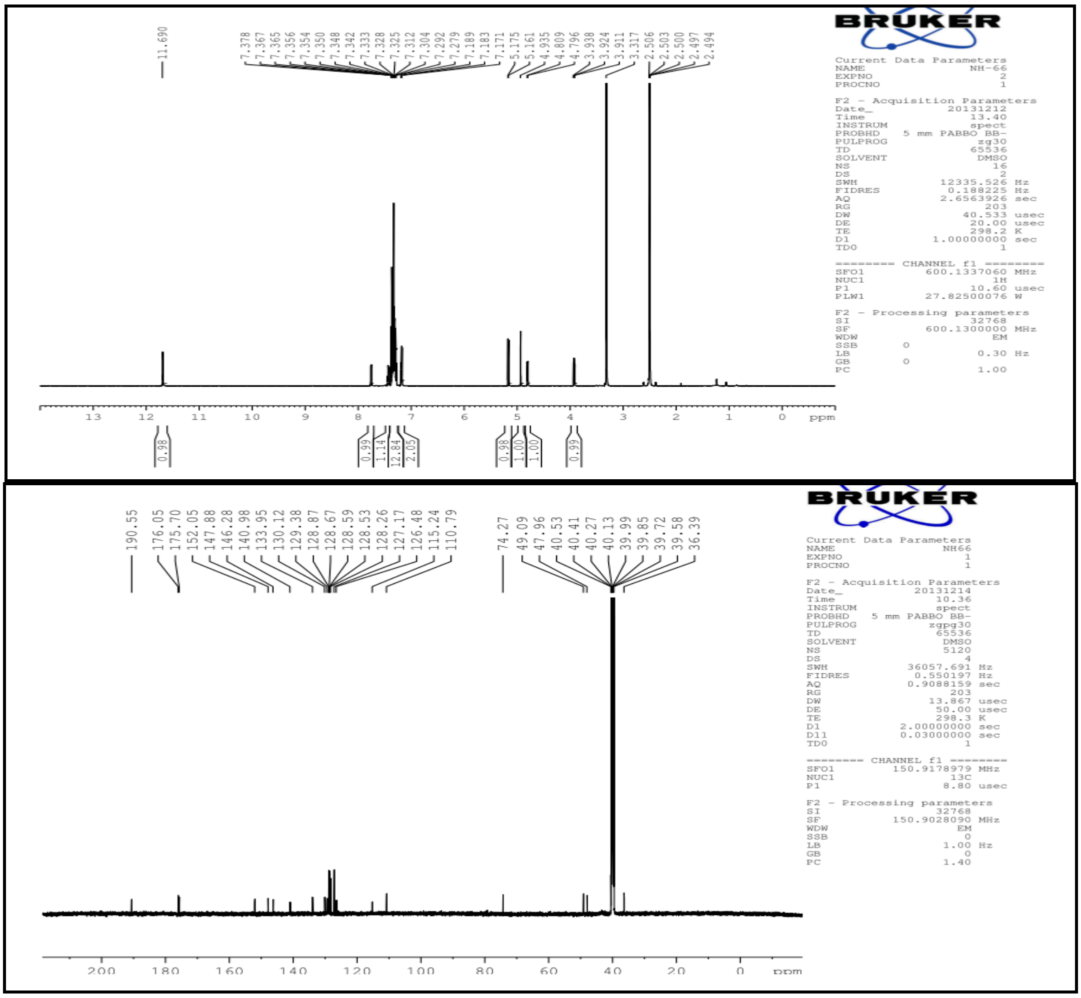

Figure S16. ^{1}H-NMR and ^{13}C-NMR spectra of compound **6a**.
